# COAPT Trial at 5 Years: Same Doubts Remain About the Efficacy of Transcatheter Edge-to-Edge in Functional Mitral Regurgitation

**DOI:** 10.21470/1678-9741-2023-0360

**Published:** 2024-10-14

**Authors:** Ovidio A. García-Villarreal, Du Chunming

**Affiliations:** 1 Mexican College of Cardiovascular and Thoracic Surgery, Mexico City, Mexico; 2 Department of Cardiac Surgery, Bozhou Hospital, Anhui Medical University, Bozhou City, Anhui, People’s Republic of China

Functional mitral regurgitation (FMR) is the result of tridimensional distortion in spatial configuration of the mitral valve (MV), whose constituent elements remain organically intact. It results from an imbalance between the forces of tension and coaptation in the opening and closing mechanism of the MV. As such, FMR is the effect but not the cause of the underlying disease. Guideline-directed medical therapy (GDMT) or myocardial revascularization, as well as cardiac resynchronization when appropriate, are the only first-line treatments to be used in FMR. Transcatheter edge-to-edge mitral valve repair (M-TEER) has emerged as one of the latest cutting-edge breakthroughs for non-surgical cases with FMR. The Cardiovascular Outcomes Assessment of the MitraClip Percutaneous Therapy for Heart Failure Patients with Functional Mitral Regurgitation (COAPT) trial is the only randomized controlled trial (RCT) supporting the use of M-TEER in FMR.

The five-year results of the COAPT trial have been recently released^[1]^. In this RCT, 302 patients with severe FMR who underwent M-TEER in addition to GDMT were compared to 312 who received GDMT alone. The primary effectiveness endpoint was measured by all hospitalizations for heart failure (HF) rate, which was 61% *vs.* 83%, respectively (hazard ratio [HR], 0.49; 95% confidence interval [CI], 0.40-0.61). Additionally, death for any cause was 57.3% *vs.* 67.2%, respectively (HR, 0.72; 95% CI, 0.58 to 0.89), and death for any cause in combination with hospitalization for HF was 73.6% *vs.* 91.5%, respectively (HR, 0.53; 95% CI, 0.44 - 0.64).

In general terms, HF hospitalization and death for any cause rates improved in favor of M-TEER plus GDMT over GDMT alone at five years. However, these facts need to be carefully clarified.

Firstly, the prevailing management attitudes today for FMR are based on the notion of “FMR elimination” which is inappropriate by scientific principles, since FMR is the effect but not the cause of the primary disease. Thus, due to a lack of basic information and the usage of crudely conceived models, the predictive capabilities of these trials can often be erroneous. The situation requires close scrutiny to understand that several critical findings are lacking.

When comparing echocardiographic baseline values and five years after M-TEER, statistical difference was seen only in MV orifice area, MV gradient, and right ventricular systolic pressure. All the remainder, including regurgitant volume, regurgitant fraction, left ventricular (LV) end-systolic diameter, LV end-systolic volume, LV end-diastolic diameter, LV end-diastolic volume, LV ejection fraction, and forward stroke volume did not show any statistical difference in the device group (Table 1). This fact is extremely important since it is not in line with the expected pathophysiology of mitral regurgitation (MR).

**Table 1:** Comparison of echocardiographic data in the device group between baseline at the beginning of the enrollment and five years of follow-up.

Variable	Echocardiographic values		95% CI	*P*-value
	Basal	5 years		
LVEDV (mL)	194 ± 69.2	174 ± 67.1	-44.12 to 3.3	0.09
LVESV (mL)	135.5 ± 56.1	129 ± 65.4	-26.19 to 13.19	0.51
LVEDD (cm)	6.17 ± 0.73	6.09 ± 0.90	-0.29 to 0.13	0.46
LVESD (cm)	5.28 ± 0.86	5.32 ± 1.02	-0.22 to 0.28	0.81
LVEF (%)	31.3 ± 9.1	28.3 ± 11.3	-6.22 to 0.22	0.07
Regurgitant volume (mL)	28.8 ± 17	21.1 ± 10.1	-19.07 to 3.67	0.18
RF (%)	38.2 ± 13.8	37.8 ± 12.8	-9.78 to 8.98	0.93
RVSP (mmHg)	44.0 ± 13.4	34.9 ± 12.9	-13.26 to -4.93	< 0.0001
Forward stroke vol. (mL)	50.5 ± 16.5	49.7 ± 18.41	-6.05 to 4.45	0.76
MV orifice area (cm^2^)	5.17 ± 1.26	3.76 ± 1.11	-2.17 to -0.64	0.0003
MV gradient (mmHg)	2.5 ± 1.1	4.1 ± 2.3	1.16 to 2.03	< 0.0001
EROA (cm^2^)	0.41 ± 0.15	0.15 ± 0.05	-0.37 to -0.14	< 0.0001

CI=confidence interval; EROA=effective regurgitant orifice area; LVEDD=left ventricular end-diastolic dimension; LVEDV=left ventricular end-diastolic volume; LVEF=left ventricular ejection fraction; LVESD=left ventricular end-systolic dimension; LVESV=left ventricular end-systolic volume; MV=mitral valve; RF=regurgitant fraction; RVSP=right ventricular systolic pressure

The COAPT trial reported the forward stroke volume after five years of M-TEER as 49.7 ± 18.41 mL. Nevertheless, if the mean values reported in the supplementary material are taken to calculate the hypothetical forward stroke volume - LV end-diastolic volume (174 mL) minus LV end-systolic volume (129 mL) and minus regurgitant volume (21.1 mL) -, the theoretically calculated stroke volume is 23.9 mL. If cardiac outputs were calculated with a hypothetical heart rate of 80 beats per minute and 1.7 m2 of body surface area, then the result would be 1.9 Lt/min, with a cardiac index of 1.1 Lt/min/m2, which would be incompatible for a patient integrated into activities of daily life outside the hospital. While the limitations of MR measurement by echocardiography may have contributed to this result, it is important to address and resolve any issues with inaccurate measurement of MR in order to ensure accurate judgment of endpoint events. This responsibility falls on the investigators of the COAPT clinical trial and other relevant researchers^[2]^.

In a detailed scrutiny by comparing the results at five years between the device and control groups, no statistical difference was identified between them (Table 2). In this context, the trial has the negative connotation to fail in demonstrating a clear and logical benefit of M-TEER in terms of pathophysiologic status.

**Table 2:** Comparison of echocardiographic data at five years of follow-up between device and control groups.

Variable	Echocardiographic values at 5 years		95% CI	*P*-value
	Device group	Control group		
LVEDV (mL)	174 ± 67.1	166.6 ± 77	-42.02 to 27.22	0.67
LVESV (mL)	129 ± 65.4	119.3 ± 70.6	-42.39 to 22.99	0.56
LVEDD (cm)	6.09 ± 0.90	5.97 ± 0.91	-0.47 to 0.23	0.51
LVESD (cm)	5.31 ± 1.02	5.15 ± 1.02	-0.57 to 0.26	0.45
LVEF (%)	28.3 ± 11.3	32.2 ± 14	-2.18 to 9.98	0.20
Regurgitant volume (mL)	21.1 ± 10.1	18.8 ± 6	-10.60 to 6.00	0.56
RF (%)	37.8 ± 12.8	32.8 ± 9.2	-16.14 to 6.14	0.35
RVSP (mmHg)	34.9 ± 12.9	35.4 ± 12.1	-5.25 to 6.25	0.86
Forward stroke vol. (mL)	49.7 ± 18.41	47.6 ± 16.8	-9.78 to 5.58	0.59
MV orifice area (cm^2^)	3.76 ± 1.11	4.33 ± 1.31	-0.51 to 1.65	0.28
MV gradient (mmHg)	4.1 ± 2.3	3.5 ± 4.3	-2.00 to 0.80	0.40
EROA (cm^2^)	0.15 ± 0.05	0.15 ± 0.06	-0.06 to -0.14	1.000

CI=confidence interval; EROA=effective regurgitant orifice area; LVEDD=left ventricular end-diastolic dimension; LVEDV=left ventricular end-diastolic volume; LVEF=left ventricular ejection fraction; LVESD=left ventricular end-systolic dimension; LVESV=left ventricular end-systolic volume; MV=mitral valve; RF=regurgitant fraction; RVSP=right ventricular systolic pressure

It is worth noting the extremely low percentage of patients in which the results of five-year echocardiographic study were reported. According to supplementary material, 79 patients were at risk of death for any cause at five years after M-TEER. However, the assessment for regurgitant volume as well as regurgitant fraction was performed only in nine cases (11.3%). Even worse is identified in the case of effective regurgitant orifice area, whose calculation was performed only in seven cases (8.8%). Moreover, if the sample size is calculated by using a CI of 95%, marginal error of 5%, and population size of 79, then the minimum sample size to be statistically reliable is 66 patients, but not nine or seven, as it was calculated in COAPT at five years. Consequently, one should approach with some skepticism the reported benefits in favor of M-TEER in COAPT until underlying assumptions are well clarified.

Having addressed these concerning points, attention must be focused now on the GDMT used in the COAPT trial. Much discussion has been centered on the use of M-TEER for FMR in non-responders to GDMT. However, models using an optimal, true, and sole GDMT have been quite useful in understanding and assessing the behavior of the FMR without the need for M-TEER. According to the recommendations by the current clinical guidelines for the management of patients with heart failure with reduced ejection fraction (HFrEF), the use of quadruple therapy consisting of beta-blockers (BB), mineralocorticoid receptor antagonists (MRA), angiotensin receptor-neprilysin inhibitors (ARNi), and sodium-glucose cotransporter-2 inhibitors (SGLT2i) at optimal doses is essential^[3]^. This is especially important given the current evidence in favor of the beneficial effect of sacubitril/valsartan (ARNi) in patients with HFrEF in FMR^[4]^. In the Prospective Study of Biomarkers, Symptom Improvement, and Ventricular Remodeling During Sacubitril/Valsartan Therapy for Heart Failure (PROVE-HF), approximately 50% of cases with severe FMR shifted to non-severe FMR with the sole use of ARNi after 12 months of treatment at optimal doses^[5]^. In another study by Spinka et al.^[6]^, GDMT titration was associated with a reduction of 39.3% in the severity of FMR, nearly a three-fold benefit of such a reduction (odds ratio, 2.91; 95% CI, 1.34 to 6.32; *P*=0.007). In addition, significative reverse remodeling and clinical improvement were also observed.

In the COAPT at five years, BB were administered in 92/108 (85.2%) patients of the device group and 70/79 (88.6%) patients of the control group (95% CI, -7.02 to 12.9, *P*=0.50); for diuretics, 90/108 (83.3%) *vs.* 65/79 (82.3%) (95% CI, -9.6 to 12.5, *P*=0.86), respectively; for MRA (spironolactone), 56/108 (51.9%) *vs.* 37/79 (46.8%) (95% CI, -9.2 to 19.1%, *P*=0.49), respectively; and for ARNi (sacubitril/valsartan), 42/108 (38.9%) *vs.* 18/79 (22.8%), respectively (95% CI, 2.6 to 28.4; *P*=0.02). No case of SGLT2i administration was reported in both groups.

An overriding concern is the fact that, despite no significant difference between groups for any of the items abovementioned, there are low percentages of ARNi administration in the device group, this factor being more prevalent in the GDMT group. However, if the total numbers are analyzed, of 312 patients initially enrolled, only 18 (18/312= 5.7%) received sacubitril/valsartan at five years in the control group (GDMT alone), compared with 42 of 302 cases (14 %) in the device group (M-TEER+GDMT) (95% CI, 3.6 to 13.1; *P*<0.001). Studies have shown that MRAs are well tolerated even in patients with advanced HFrEF^[7]^. In the COAPT trial, only about half of the patients used MRAs, both for the device and GDMT groups, which may be inadequate. In the Safety, Tolerability and Efficacy of Up-Titration of Guideline-Directed Medical Therapies for Acute Heart Failure (or STRONG-HF) trial, also applied to patients with advanced HFrEF, all three groups composed of BB, angiotensin-converting enzyme inhibitors (ACEI)/angiotensin receptor blockers (ARB)/ARNi, and MRA achieved > 90% drug utilization^[8]^.

From these observations, some special flaws must be outlined. Since raw data for this trial are not publicly available, the total number of patients receiving ARNi/MRA/BB is unknown. The fact that the most frequently used drugs on this trail were BB and diuretics is too worrying. That is, with this poor and low quality GDMT, it is extremely difficult to find “good responders” to medical treatment, regardless of the assigned group. In other words, it would be a mistake to think that FMR could be treated only with BB and diuretics.

As for the GDMT adherence in real world, a low rate of adherence in eligible patients has been observed. Komajda et al.^[9]^ reported that less than 25% adhere to guidelines recommendations. In addition, according to the Change the Management of Patients with Heart Failure (CHAMP-HF) Registry, in patients eligible for multiple GDMT, only 1% received target doses of ACEI/ARB/ARNi/BB, and MRA^[10]^. These considerations are of paramount importance since they take place in RCTs, such as COAPT. As a matter of fact, it has been found that only 2.2% of patients in the COAPT trial received a GDMT protocol at optimal doses^[11]^.

In summary, the GDMT leaves much to be desired in the five-year COAPT trial, especially in the GDMT control group. Obviously, much effort must be directed to improve these models when planning a RCT. A more sensible view related to a true optimal and efficient use of a contemporary GDMT is required. At present, with data available coming from COAPT at five years is not possible to reach any definite conclusion.

To draw a general conclusion, one must consider specific factors as well as their interrelationships. However, this is actually of great difficulty, since the broad spectrum of these factors is given through incomplete versions of them, resulting in much ambiguity.

Thus, the main concern is the fact that COAPT aims to illustrate how FMR behaves in relation to each of the analyzed factors, based upon an inadequate and unrealistic GDMT.

Erroneous or unrealistic scenarios have developed our current understanding of M-TEER. In such a way that too often we overlook the most valuable parts when trying to get rid of something unwanted in search of answers compatible with our shortage of knowledge. Models and decisions may be erroneously inappropriate to be applied in real-life FMR cases. There has been a tendency to rely on partial approaches rather than fully treating the basic underlying causes. It does not appear that there is any one single, all-encompassing solution to improving FMR. However, the pivotal importance of ensuring optimal GDMT before M-TEER should be stressed. All things considered, another new and much more realistic RCT is urgently needed in order to get a true update in the way we evaluate the management of FMR. This necessarily implies dissemination of reliable facts derived from transparent and intelligent RCTs. In so doing, the net effect should be a significant impact in improving the management of FMR.
